# A C-Repeat Binding Factor Transcriptional Activator (CBF/DREB1) from European Bilberry (*Vaccinium myrtillus*) Induces Freezing Tolerance When Expressed in *Arabidopsis thaliana*

**DOI:** 10.1371/journal.pone.0054119

**Published:** 2013-01-17

**Authors:** Rachael J. Oakenfull, Robert Baxter, Marc R. Knight

**Affiliations:** Durham Centre for Crop Improvement Technology, Durham University, Durham, United Kingdom; 2 School of Biological and Biomedical Sciences, Durham University, Durham, United Kingdom; Kansas State University, United States of America

## Abstract

Freezing stress affects all plants from temperate zones to the poles. Global climate change means such freezing events are becoming less predictable. This in turn reduces the ability of plants to predict the approaching low temperatures and cold acclimate. This has consequences for crop yields and distribution of wild plant species. C-repeat binding factors (CBFs) are transcription factors previously shown to play a vital role in the acclimation process of *Arabidopsis thaliana*, controlling the expression of hundreds of genes whose products are necessary for freezing tolerance. Work in other plant species cements CBFs as key determinants in the trait of freezing tolerance in higher plants. To test the function of CBFs from highly freezing tolerant plants species we cloned and sequenced CBF transcription factors from three *Vaccinium* species (*Vaccinium myrtillus, Vaccinium uliginosum* and *Vaccinium vitis-idaea*) which we collected in the Arctic. We tested the activity of CBF transcription factors from the three *Vaccinium* species by producing transgenic *Arabidopsis* lines overexpressing them. Only the *Vaccinium myrtillus* CBF was able to substantially activate *COR* (CBF-target) gene expression in the absence of cold. Correspondingly, only the lines expressing the *Vaccinium myrtillus* CBF were constitutively freezing tolerant. The basis for the differences in potency of the three *Vaccinium* CBFs was tested by observing cellular localisation and protein levels. All three CBFs were correctly targeted to the nucleus, but *Vaccinium uliginosum* CBF appeared to be relatively unstable. The reasons for lack of potency for *Vaccinium vitis-idaea* CBF were not due to stability or targeting, and we speculate that this was due to altered transcription factor function.

## Introduction

Freezing stress affects all plants from temperate zones to the poles. The incidence of freezing stress is largely predictable: generally ensuing at specific times of the year. However, the strength of this prediction is now being eroded by climate change, which is leading to more chaotic patterns of climate, including unexpected cold episodes *e.g.* during the summer [1], as well as extreme winter temperature events due to altered patterns of landscape snow cover [2]. This is becoming a serious agricultural problem *e.g.* in recent years there have been a number of examples worldwide of crops being lost to freezing temperatures. Multiple states in the USA lost fruit crops in April 2012 [3]. In 2011 Mexico lost 90% of its corn crops to frost [4], which in turn caused food prices in that country to triple that year. In the same year India had its coldest winter in 30 years [5] and Zimbabwe (a major exporter of flowers) lost a large number of flower crops to frost [6]. Such problems arise because levels of freezing tolerance of plant/crop species are not fixed, but require an active process known as cold acclimation, which needs to be timed to precede freezing events.

Temperate and Arctic plants usually survive winter by cold acclimating, triggered by temperatures slowly becoming lower and the day length decreasing. This combination of light and temperature acts as a signal to the plant that freezing events are likely in the near future. Depending on the plant species, the acclimation process can take from a few days to several weeks to complete. Acclimation involves metabolic and cellular changes to allow survival of freezing temperatures, including the production of compatible solutes and reconfiguration of membrane components [7]. The majority of these responses are mediated by changes in gene expression during the acclimation process.

In the model plant system *Arabidopsis*, key players in this molecular response are the CBF/DREB1 (C-repeat Binding Factor/Drought Response Element Binding Factor) transcription factors [8]. These transcription factors are induced in expression in response to low, non-freezing temperatures. These transcription factors in turn activate the expression of hundreds of genes (*COR* (cold-regulated) genes) whose products effect the changes needed for cold acclimation. In most species tested, CBFs occur as multigene families. In the case of *Arabidopsis*, the family of CBFs which regulates cold gene expression total three members [9]. Reports are mixed in terms of equivalence of activity, with most studies concluding they have equal function *e.g.* [10] although there is some evidence in *Arabidopsis* of a hierarchy of regulation, whereby CBF2 acts as a negative regulator of CBF1 and CBF3 [11], [12]. Studies of other species of plants strongly support the importance of CBF/DREB1 transcription factors in cold acclimation of many species, including the genus *Vaccinium* [4], [13]. The importance of CBF/DREB1 to freezing tolerance can be demonstrated by overexpressing these transcription factors, which leads to constitutive expression of *COR* genes and constitutive freezing tolerance, in the total absence of physiological cold acclimation. Recently, overexpression of *Vaccinium corymbosum* CBF in *Arabidopsis* was demonstrated to lead to induced *COR* expression, and constitutive freezing tolerance [13], indicating a functional CBF system in this closely related *Vaccinium* species. Additionally, overexpression of CBF from *Vaccinium corymbosum* in transgenic native background (same species) was demonstrated to lead to increased freezing tolerance [14]. This study demonstrates that CBF genes are indeed functional in a *Vaccinium* background in achieving freezing tolerance. This has also been demonstrated for other plant species *e.g. Brassica napus* [4]. Natural variation in CBF function has been postulated to be a substrate for selection by evolution *e.g.* the Cape Verde accession of *Arabidopsis* has a greatly reduced CBF function and is found in a tropical climate. Another important aspect of the cold acclimation response is developmental, i.e. there is altered (reduced) growth. This has been shown to be, at least partially, mediated by the DELLA growth-regulation system i.e. DELLA protein levels accumulate at cold temperatures, leading to reduced growth. Interestingly, CBF/DREB1 have also been shown to be important in regulating this process, by suppressing flux through the gibberrellin pathway, which promotes stability of DELLA proteins [15].

We were interested in assessing the potency of CBF/DREB1 transcription factors from three plant species from the genus *Vaccinium*, namely *V*. *myrtillus*, *V. uliginosum* and *V. vitis-idaea* that are key components of circumpolar Arctic tundra heath communities [16], [17], [18]. In such communities, these species have to tolerate significantly low temperatures, often for extended periods of the yearly growth cycle.

## Materials and Methods

### Plant Materials and Sampling Locations

*V. myrtillus*, *V. uliginosum* and *V. vitis-idaea* samples were collected in Abisko, Sweden (68°20’N 18°49’E), 400 m above sea level and 50 km from the nearest sea coast [19]. Temperatures fluctuate between −35°C and +18°C over the year.

Seeds for *Arabidopsis thaliana* accession Columbia (Col-0) were obtained from Lehle seeds (Round Rock, Texas, USA). Seeds for *Nicotiana benthamiana* were a kind gift from Dr Tim Hawkins (Durham University, UK).

### Plant Growth Conditions

Sterilised *Arabidopsis* seeds were grown on 1× MS medium, 0.8% (w/v) agar plates [20] and grown at a temperature of 20°C and 16/8 h photoperiod with a light intensity of 150 µmol m^−2^ s^−1^ in a Percival plant growth cabinet (Model: CU-36L5D, CLF plant climatics, Emersacker, Germany) for 7 days. After this time seedlings were transferred to peat plugs (Jiffy products international AS, Norway) and grown in a bespoke walk-in growth chamber with approximately the same conditions. *Nicotiana benthamiana* was grown in a similar bespoke walk-in growth chamber at a daytime temperature of 24°C and a night time temperature of 21°C, 16/8 h photoperiod with a light intensity of approximately 200 µmol m^−2^ s^−1^.

### Genomic DNA Extraction, Sequencing and Analysis

DNA was extracted from *Vaccinium* leaf tissue using the CTAB DNA extraction method [21] with an added phenol-chloroform clean-up stage: this involved resuspending in phenol chloroform (1∶1 DNA aqueous solution to phenol chloroform (v/v)) followed by centrifugation, removal of the aqueous top layer and repeating until no more precipitate was formed at the interface between the aqueous top layer and phenol chloroform bottom layer. DNA was precipitated by adding one tenth of the volume of the aqueous layer of 3 M sodium acetate (pH 4.5) and two volumes of 100% ethanol at −80°C. The pelleted DNA was washed in 1 ml of 80% ethanol and resuspended in TE buffer. CBF coding regions were amplified by PCR using RedTaq (Bioline Reagents Ltd, London, UK) and the CBF-VI primers listed below. PCR products were separated by TBE-agarose gel electrophoresis, cut-out of the gel and purified using a Qiaquick gel extraction kit (Qiagen Ltd. Crawley, UK). Sequencing was performed by the Durham University sequencing service. Sequences were aligned using Clustal X and Jalview [22], [23]. Sequences for the *Vaccinium myrtillus*, *Vaccinium uliginosum* and *Vaccinium vitis-idaea* DREB1 cDNA clones were submitted to GenBank (accession numbers, JN254610, JN866911 and JN866912, respectively).

### Vaccinium RNA Extraction

Leaf tissue from the three *Vaccinium* species was cold treated on ice for two hours with an ambient room temperature control. Total RNA was extracted using the method described in Jaakola et al., 2001 [24].

### Production of Plant Transformation Constructs

For overexpression of *Vaccinium* CBFs (vCBFs) in *Arabidopsis*, CBF coding regions were amplified from *Vaccinium* genomic DNA using the following primers: CBF-VI-R: 5′-ATCTAACTCCACAAGAGACC-3′ and CBF-VI-F: 5′-CACCGAAGAGTTAGATGTGCGCAGC-3′ which amplify sequences from all three *Vaccinium* species. Sequences were amplified and sequenced on two separate occasions to ensure that the correct sequence was obtained without PCR errors. The resulting PCR products were cloned into pENTR-D-TOPO (Invitrogen) and then into the PB7WG2 destination binary vector [25] using a Gateway LR Clonase II kit (Invitrogen). This produced constructs where each of the three *Vaccinium* CBF coding regions were inserted after the 35S promoter for overexpression. Each CBF coding region started at the start codon and ended at the stop codon, so the constructs did not have any native CBF 5′ or 3′ untranslated sequences. In this way the constructs were only different to each other in the CBF coding region itself, allowing for proper comparison between the activity of each CBF protein when expressed. For production of GFP-CBF fusions, vCBF coding regions were amplified using the following primers: Myrtillus CBF-F: 5′-CACCATGGAATATTACTCAAGTCC-3′ or Uli-vitis- CBF-F: 5′-CACCATGGAATATAACTCTAGTCC-3′ as forward primers and CBF-VI-R: 5′- ATCTAACTCCACAAGAGACC-3′ as reverse primer. PCR products were cloned into pENTR-D-TOPO (Invitrogen, Life technologies Ltd. Paisley UK) then into the pK7WGF2 destination binary vector [25] using LR Clonase and a DH5α *E. coli* strain. After completion of binary constructs, the constructs were sequenced to confirm there were no errors and, where relevant, that the CBF coding regions were in frame with GFP. The binary vectors also conferred resistance to BASTA to allow for selection of transgenic lines (see below). For transactivation studies in *Nicotiana benthamiana*, a binary construct to express firefly luciferase driven by 4 copies of the CRT/DRE promoter motif [26] was used in conjunction with the GFP constructs.

### Plant Transformation

For stable transformation of *Arabidopsis*, *Arabidopsis* Col-0 *was* transformed by *Agrobacterium*-mediated floral dip [27] using *Agrobacterium tumefaciens* strain C58C1. Transformants were grown on a mixture of sand and Perlite and selected by spraying the seedlings from 7 days onwards with 150 µM BASTA (herbicide) at 5 day intervals. Individual plants were bulked up and segregating M1 seed used to grow seedlings for gene expression experiments, and M2 homozygotes selected for freezing and growth assays. For transient expression of *Nicotiana benthamiana*, *Agrobacterium tumefaciens* infiltration method was used [28]. Bacteria were resuspended in 10 mM MgCl_2_ and infiltrated at an OD of 0.3. Plants were grown for a further for 2 days before imaging.

### Fluorescence and Luminescence Imaging

GFP fluorescence was imaged using a Zeiss Meta 510 CLSM (confocal laser scanning microscope) and software at a magnification of x40 (oil immersion lens). Luciferase imaging was performed after spraying leaves with 5 mM potassium luciferin in 0.01% Triton X-100 (v/v) using a Photek photon-counting camera (Photek, Hastings, UK) [29].

### Western Blot Analysis

Total protein was extracted from *Nicotiana benthamiana* leaf disks (as described in [30]) using a BioRad D_c_ Protein assay. 50 ng of the extracted protein was boiled in SDS loading buffer and run on a 15% polyacrylamide gel for ∼3 hours. Proteins were then transferred onto PVDF membrane (BioRad laboratories Ltd. Hertfordshire, UK) using a semi-dry blotting technique. The membrane was then blocked in 5% milk powder in TBST and incubated with a 1 in 200 dilution of αGFP antibody (#G1112 Santa Cruz Biotechnology inc. California, USA) in 10% (w/v) milk powder in TBST overnight at 4°C. Anti-rabbit IgG HRP secondary antibody (#E1012 Santa Cruz Biotechnology inc. California, USA) was incubated for 1 hour at room temperature as a 1 in 5000 dilution in 5% (w/v) milk in TBST. After this, the filter was then washed and incubated with a WestDura Femto ECL kit (Fisher Scientific, Leicestershire, UK) and bands visualised using a photo counting camera (Photek, Hastings, UK).

### qPCR Analysis of Gene Expression

Quantitative real-time PCR was use to analyse gene expression levels as described previously [31]. cDNA was synthesised from *Arabidopsis* RNA using a high capacity cDNA reverse transcription kit (Applied Biosystems, California, USA). cDNA was synthesised from *Vaccinium* RNA using a QuantiTect reverse transcription kit (Qiagen, Sussex, UK). qPCR was carried out using an AB 7300 real time PCR system (Applied Biosystems California USA) and Go Taq qPCR master mix (Promega, Wisconsin,USA). Primers were designed by eye and synthesised by Invitrogen. Sequences used: Vaccinium Dreb1 Reverse: 5′-AAGAAGGCGAGGGAGAGAGT-3′ and Dreb1 Forward: 5′-GGGAATGGGAGTGAGGTTTT-3′; KIN2 Forward: 5′-CTGGCAAAGCTGAGGAGAAG-3′ and KIN2 Reverse: 5′-ACTGCCGCATCCGATATACT-3′; COR15a Forward: 5′-CGTTGATCTACGCCGCTAAAG-3′ and COR15a Reverse: 5′-CTACACCATCTGCTAATGCC-3′; COR414 Forward: 5′-GGGAGAGTATGGTGTATGGGCA-3′ and COR414 Reverse: 5′- TGATATGGCGCCACAATCA-3′; GOLS3 forward: 5′-CAAAGTTGTCCCTCCCACAC-3′ and GOLS3 Reverse: 5′-GAGCATGGCCAAGACAAGAT-3′; *Arabidopsis* endogenous control: PEX4 Forward: 5′-TCATAGCATTGATGGCTCATCCT-3′ and PEX4 Reverse-ACCCTCTCACATCACCAGATCTTAG-3′. *Vaccinium* endogenous control: Tubulin Forward : 5′-AGGAAATGTTTAGGCGTGTGAGC-3′ and Tubulin Reverse 5′-AGTGAACTCCATCTCGTCCATACC-3′. Error bars shown in Figures depicting qPCR data represent RQ_MIN_ and RQ_MAX_ and constitute the acceptable error level for a 95% confidence level according to Student’s t-test.

### Freezing Assays

Transgenic *Arabidopsis* lines overexpressing vCBFs were tested for *vCBF* and *COR* gene expression in 4 independent lines for each construct. Two transgenic lines produced from using each of the three different *Vaccinium* constructs were chosen for freezing assays: one showing relatively high expression, the other relatively low expression, giving 6 lines in total. Seeds from each of the 6 chosen lines and Col-0 wild type control were germinated and grown for 5 weeks (in the same way as described above) then transferred straight to −7°C for 24 h in a Sanyo MIR 254 incubator in darkness. The plants were then left to thaw and returned to their original growth conditions. Before being transferred to the freezing chamber each plant was individually photographed (using a Cannon EOS 550D DSLR on Raw format to produce TIFF images), then photographed again 3 days after freezing. The average diameter of each plant rosette was calculated from 3 measurements between the points of widest diameter using AxioVison software (Carl Zeiss, Cambridge, UK) and then standardised between photos using the scale bar in the corner of each photo (not shown).

### F_v_/F_m_ Measurements

Damage due to stress can be diagnosed as a reduced ratio of variable fluorescence (F_v_) over maximal fluorescence (F_m_) of plant photosystems [32], [33]. To test the effect of freezing on plants lines we therefore determined F_v_/F_m_. Before being transferred to the freezing chamber the F_v_/F_m_ value for each plant was measured using a FluorCam 700 mf (Photon Systems instruments, Brno, Czech Republic) on the F_o_, F_m_ and Kautsky effect setting. Plants were dark-acclimated for 30 min prior to measurements. Measurements were repeated 3 days after freezing using the same parameters for F_o_ and F_m_ measurement, Kautsky induction, and dark relaxation before and after Kautsky induction.

## Results

### Protein Sequence Variation in CBF from Three Different *Vaccinium* Species

**Figure 1:**
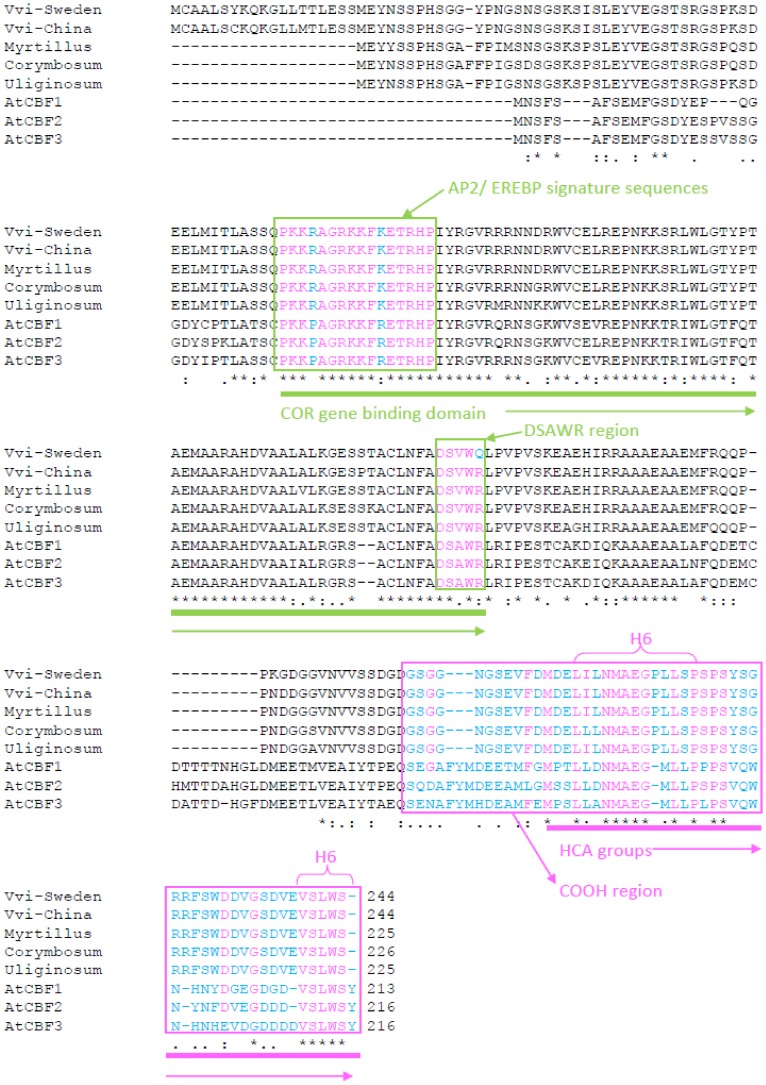
Comparison between *Vaccinium* and *Arabidopsis* CBF protein sequences. Protein lineups of *V. vitis-idaea* from both Northern Sweden and China; *V. myrtillus*; *V. corymbosum*; *V. uliginosum* and the three *A. thaliana* CBF sequences. The AP2/EREBP and DSAWR regions of the *COR* gene binding domain identified in *Arabidopsis* [40] are labelled in green boxes and thick underline along with the recognisable areas of the hydrophobic domains in the COOH region as described in *Arabidopsis* [42] labelled in a pink box and underline.

*V. myrtillus*, *V. uliginosum* and *V. vitis-idaea* are freezing-tolerant in the wild, albeit to different extents [34], [35], [36]. CBF transcription factors have been shown to be key genetic determinants of freezing tolerance in many plants [3], [4], [37]. For at least one other species of *Vaccinium*, *Vaccinium corymbosum*, it has been demonstrated that a functional CBF is present and capable of promoting freezing tolerance when overexpressed in *Arabidopsis* [13]. We wished to determine if this was the case for *V. myrtillus*, *V. uliginosum* and *V. vitis-idaea*. We first cloned and sequenced the coding region of a CBF gene from each species, utilising the sequence information already published for *V. vitis-idaea* [38]. Comparing the predicted protein sequences (Figure 1) to *Arabidopsis*, the CBF sequences from all three *Vaccinium* species were closest to the *Arabidopsis* CBF3 (DREB1A) which has been clearly demonstrated to have a role in cold acclimation [39]. Amino acid sequences in regions determined by previous reports [4], [40] to be important for either binding to DNA, or activation of transcription, were well conserved in all three *Vaccinium* species (Figure 1). Comparing the predicted CBF protein sequences for each of the three Vaccinium species we cloned with the previously reported sequences for *V. vitis-idaea* [38] and *V. corymbosum* [13] showed 9–12 amino acid differences between species and 5 between our Northern Swedish *V. vitis-idaea* CBF sequence as compared to *V. vitis-idaea* from China [38]. It was also notable that the *V. vitis-idaea* CBF which we cloned and sequenced, and that from China [38] had an extra 19 amino acid N-terminal extension, not present in either *V. myrtillus*, *V. uliginosum* or *V. corymbosum*. To test the possibility that the 3 CBF genes we cloned were involved in cold responses in the host species, we performed qPCR experiments to measure the expression of all three in response to short (2 h) exposure to cold (2 h cold is reported at the peak for CBF expression in Arabidopsis [9]). As can be seen in Figure S1, all 3 CBF genes appeared to be cold-induced. Without full genome sequence information on the three *Vaccinium* species, one cannot formally discount that primers used for qPCR might not cross-hybridise with other CBF transcripts, however, these data suggest it is possible that all three CBF genes we cloned are cold inducible in their native genetic backgrounds.

### Testing the Functionality of CBF from Three *Vaccinium* Species

**Figure 2:**
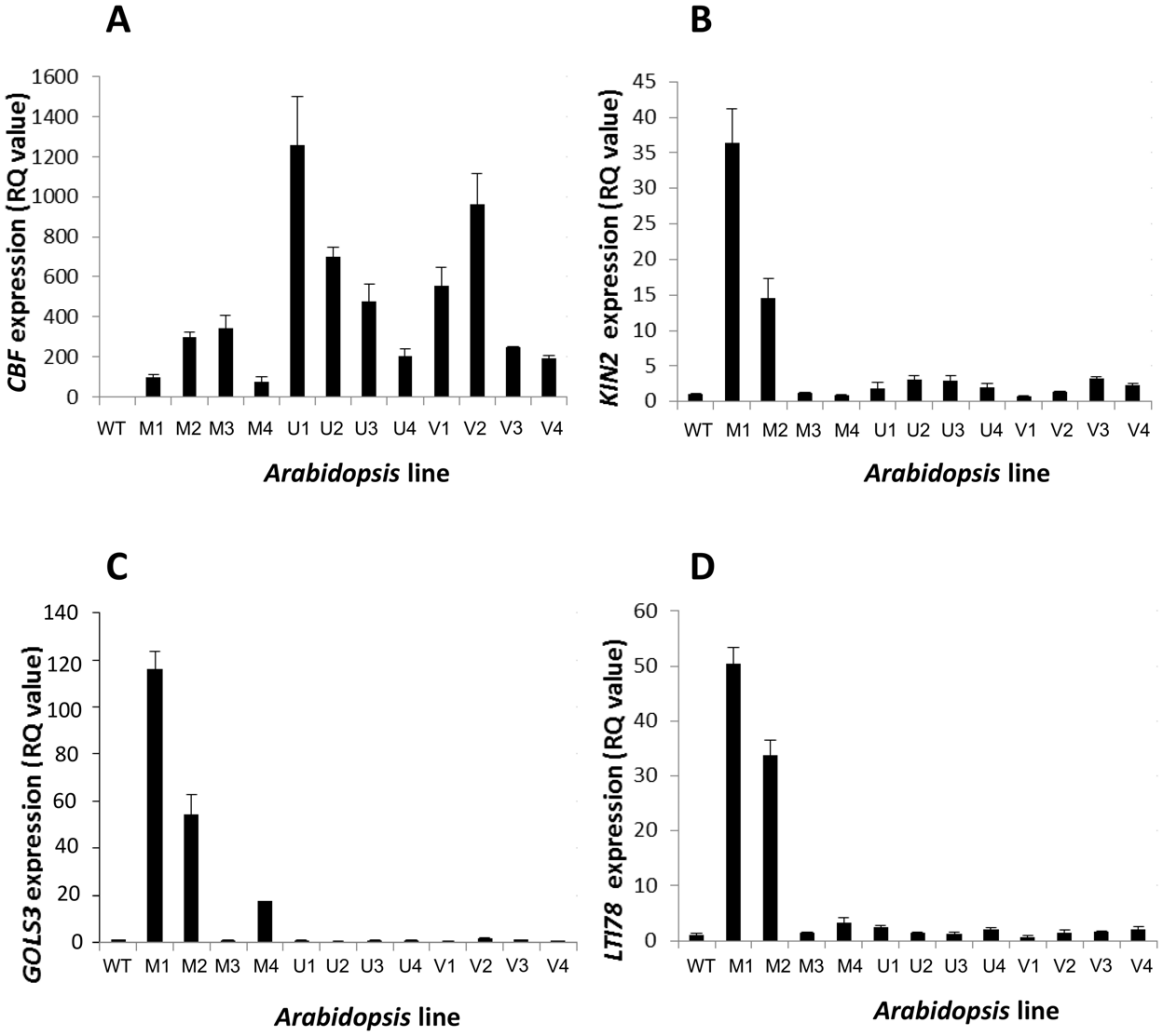
Vaccinium *CBF* and *COR* gene expression in transgenic *Arabidopsis* lines. Relative transcript abundance was measured in transgenic *Arabidopsis* lines overexpressing either *V. myrtillus*, *V. uliginosum* or *V. vitis-idaea* CBF (“M”, “U” and “I”, respectively). Four transgenic lines (labelled 1–4) for each of the three constructs were compared to wild type *Arabidopsis* (WT). Bar charts show expression of *Vaccinium CBF* (A) and *Arabidopsis KIN2* (B), *GOLS3* (C) and *LTI78* (D). Error bars represent RQ_MIN_ and RQ_MAX_ and constitute the acceptable error level for a 95% confidence level according to Student’s t-test [31].

**Figure 3:**
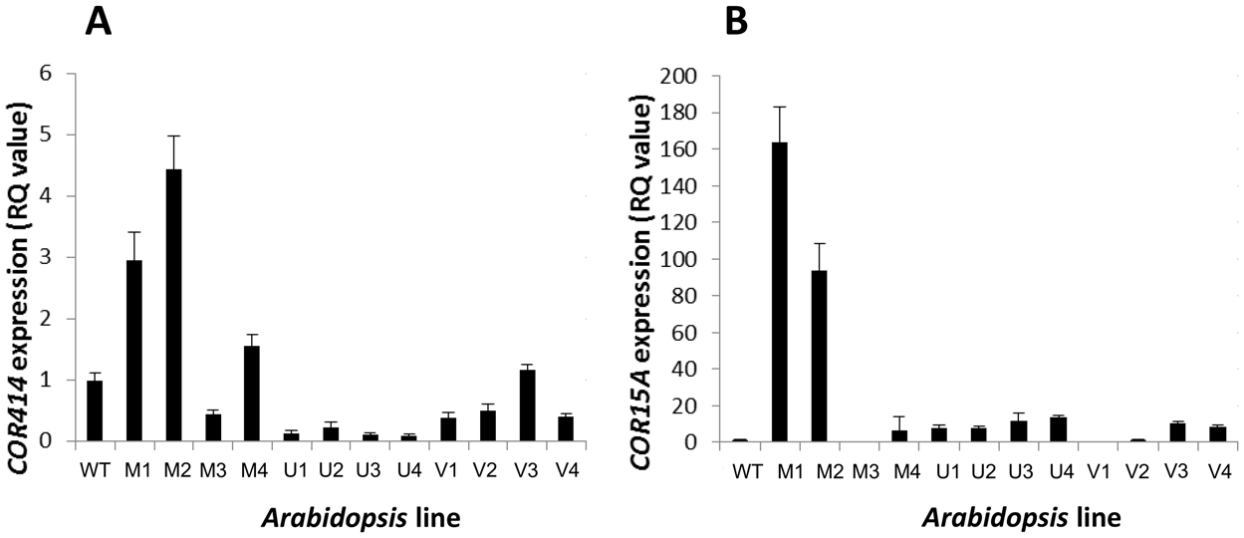
*COR414* and *COR15A* gene expression in transgenic *Arabidopsis* lines. Bar charts show expression of the *Arabidopsis COR* genes *COR414* (A) and *COR15A* (B) in the transgenic and WT *Arabidopsis* lines. Samples and details as described in legend to Figure 2.

Overexpression of functional CBF in *Arabidopsis* leads to the ectopic induction of *COR* genes, in the absence of a cold signal [37]. Therefore to test if the CBFs cloned from the three *Vaccinium* species were all functional, and whether, given the differences in protein sequence described above (Figure 1), there were differences in their activity, we expressed them all in *Arabidopsis* using genetic transformation. Lines which expressed *Vaccinium CBF* transcripts to levels detectable above background were identified using real-time PCR. Figure 2A shows typical results with 4 lines obtained from each of the three different *Vaccinium* constructs. It can be seen in Figure 2A that the levels of *CBF* transcripts achieved tended, on average, to be lower when the *V. myrtillus* sequence was expressed compared to the other 2 species (the primers and amplicon for the real time PCR were identical in all cases, so such quantitative comparisons are possible). Testing the expression of the most well-characterised CBF target genes *KIN2*, *LTI78* and *GOLS3* (Figures 2B, 2C and 2D) in the transgenic *Arabidopsis* lines demonstrated clear overexpression of all three genes in between 2–3 transgenic lines expressing *V. myrtillus* CBF. However in the other 2 types of transgenic lines (expressing *V. uliginosum* and *V. vitis-idaea* CBF) only very weak increases in expression of *KIN2* compared to wild type were observed and no significant induction of *LTI78* or *GOLS3* above wild type levels was observed. As it was reported that *V. corymbosum* CBF activated expression of *KIN2* and *LTI78* but was not able to activate expression of *COR15A* and *COR414*, we also tested the expression of *COR15A* and *COR414* in our lines overexpressing *V. myrtillus*, *V. uliginosum* and *V. vitis-idaea* CBF. Similarly to *KIN2*, *LTI78* and *GOLS3* (Figures 2B, 2C and 2D), clear overexpression of both *COR414* and *COR15A* was seen in between 2–3 transgenic lines expressing *V. myrtillus* CBF (Figure 3). In the other 2 types of transgenic lines (expressing *V. uliginosum* and *V. vitis-idaea* CBF) there was no significant induction of *COR414* (Figure 3A), but interestingly, *COR15A* was induced in all lines expressing *V. uliginosum* CBF, and 2 of the lines expressing *V. vitis-idaea* CBF (Figure 3B), relative to wild type.

### Testing Freezing Tolerance and Development in *Arabidopsis* Expressing *Vaccinium*-derived CBF

**Figure 4:**
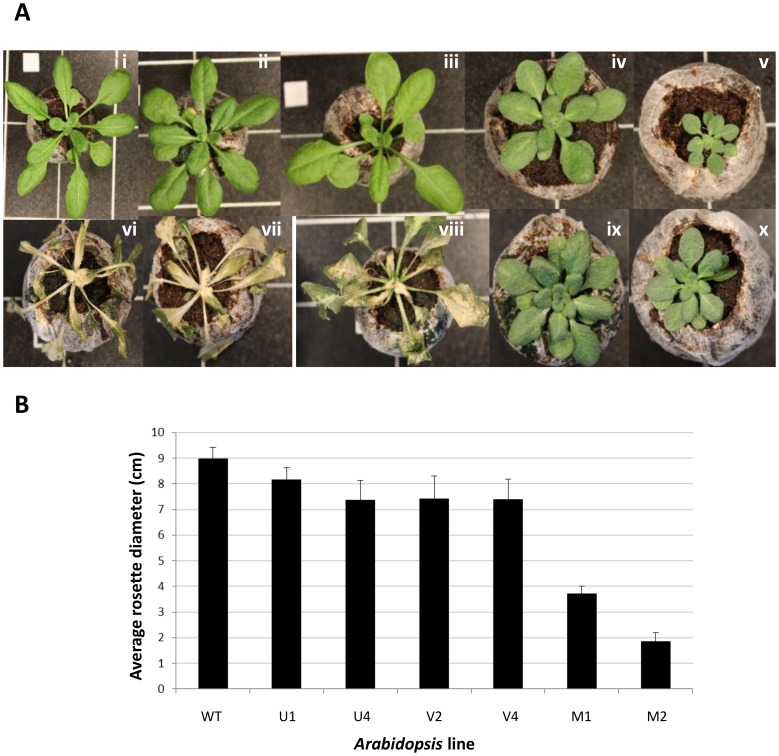
Effect of *Vaccinium* CBF overexpression upon freezing tolerance and development in transgenic *Arabidopsis*. (A) Photos show transgenic *Arabidopsis* lines expressing Vaccinium CBF: *V. myrtillus* (iv,v,ix,x); *V. uliginosum* (ii,vii); *V. vitis-idaea* (iii, viii) and wild type (i, vi), before (i-v) and after (vi-x) freezing. (B) Bar chart showing average rosette diameter of wild type (WT) *Arabidopsis* and lines overexpressing either *V. myrtillus*, *V. uliginosum* or *V. vitis-idaea* CBF (“M”, “U” and “I”, respectively), n = 8, error bars are standard errors of the mean. Transgenic line numbers correspond to those shown in Figures 2 and 3 for ease of comparison.

**Figure 5:**
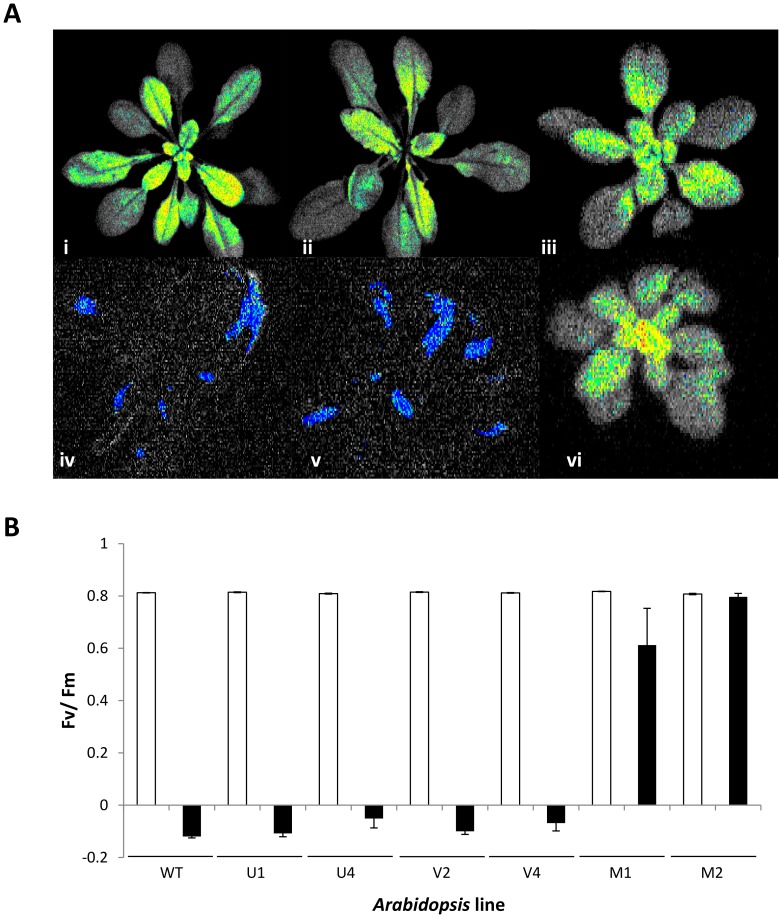
Effect of *Vaccinium* CBF overexpression upon F_v_/F_m_ after freezing. (A) Representative Fluocam images for each transgenic line measured. Before freezing F_v_/F_m_ measurement images (i-iii) for *Arabidopsis* lines expressing *V. uliginosum* (i), *V. vitis-idaea* (ii) and *V. myrtillus* (iii) CBFs. After freezing F_v_/F_m_ measurement images (iv-vi) for *Arabidopsis* lines expressing *V. uliginosum* (iv), *V. vitis-idaea* (v) and *V. myrtillus* (vi) CBFs. (B) Average F_v_/F_m_ values for each transgenic line before (white bars) and after (black bars) freezing. Each line is a transgenic *Arabidopsis* line expressing CBF from a different *Vaccinium* species: U = *V. uliginosum*, V = *V. vitis-idaea* and M = *V. myrtillus* construct, WT-wild type.

It has been demonstrated previously that overexpression of CBF in *Arabidopsis* leads to constitutive freezing tolerance, in part due to *COR* gene activation [37] and in part due to developmental growth arrest *via* DELLA proteins [41]. Given that the data from Figures 2 and 3 indicated the ability of *V. myrtillus* CBF to strongly activate the expression of *Arabidopsis COR* genes, we sought to determine whether the lines overexpressing *V. myrtillus* CBF were more freezing tolerant than lines overexpressing CBF from *V. uliginosum* and *V. vitis-idaea* and wild type. Two independent lines for each of the three overexpressing constructs were tested, along with untransformed *Arabidopsis* wild type. Individual plants were grown for 5 weeks, and then treated to a temperature of −7°C for 24 h before being returned to the original growth conditions. Figure 4 shows representative photographs of individual plants before, and after, freezing. It is clear that the transgenic lines overexpressing either *V. uliginosum* or *V. vitis-idaea* CBF were as susceptible to freezing as wild type *Arabidopsis* (Figure 4[vi] –4[viii]). However, both independent lines expressing *V. myrtillus* CBF were robustly freezing tolerant (Figures 4[ix] and 4[x]). As well as visual inspection, survival of freezing was tested by measuring Fv/Fm as an indicator of photosynthetic capacity (Figure 5). Imaging of chlorophyll fluorescence for individuals is shown in Figure 5A and average values for F_v_/F_m_ in Figure 5B. Before freezing, the F_v_/F_m_ values were very similar for all 7 genotypes. However, after freezing, it was clear that the wild type *Arabidopsis* and *Arabidopsis* expressing either *V. uliginosum* or *V. vitis-idaea* CBF suffered such severe damage that F_v_/F_m_ was unmeasurable (Figure 5B). For the 2 independent lines expressing *V. myrtillus* CBF, however, whilst there was more variability in the F_v_/F_m_ of individual plants compared to before freezing (compare size of error bars in Figure 5B), the average values were not significantly different to before freezing (Figure 5B).

The activation of *COR* genes is only one aspect of CBF function that is important for freezing tolerance, the other being DELLA-mediated grown inhibition [41]. It was clear that whilst there was little difference in overall plant size between wild type controls and lines overexpressing *V. uliginosum* and *V. vitis-idaea* CBF (Figure 4B), the lines overexpressing *V. myrtillus* showed significantly reduced diameters of rosette, as described previously when *Arabidopsis* CBF was overexpressed in *Arabidopsis* [41].

### The Molecular Basis of Potency of *V. myrtillus* CBF

It is clear from data in Figures 2–3 that even though the transcripts of *V. uliginosum* and *V. vitis-idaea* CBFs were expressed to equal (and in most cases higher) levels that *V. myrtillus* in transgenic *Arabidopsis*, target *COR* genes were not strongly activated by these two transcription factors. This leads to the question: what makes the *V. myrtillus* CBF more potent that CBFs from *V. uliginosum* and *V. vitis-idaea*? Data in Figure 2, however, do not rule out differences in protein expression, or targeting of protein to the nucleus which may account for this phenomenon.

**Figure 6:**
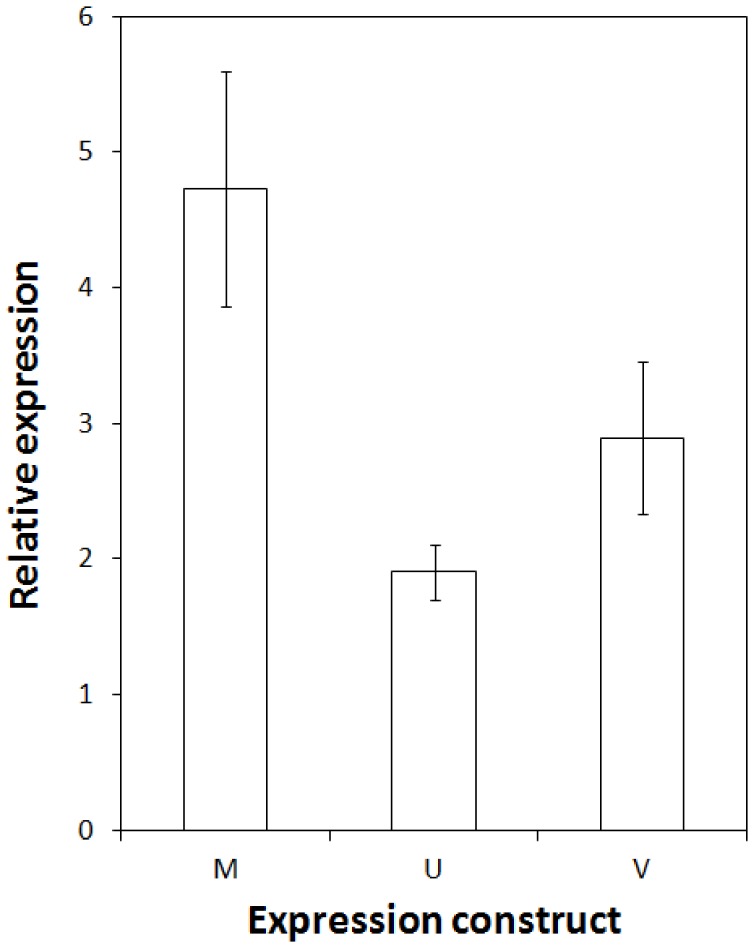
Transactivation of *CRT::LUC* by *Vaccinium* GFP-CBF fusions. Bar chart showing relative transient expression levels of *CRT::LUC* co-expressed with *Vaccinium* GFP-CBF in *Nicotiana benthamiana*. “M”, “U” and “I” denote *V. myrtillus*, *V. uliginosum* or *V. vitis-idaea* CBF, respectively. Values are ratios of LUC luminescence normalised for area, and also normalised by dividing by LUC luminescence obtained by co-expressing a free GFP control with *CRT::LUC*.

**Figure 7:**
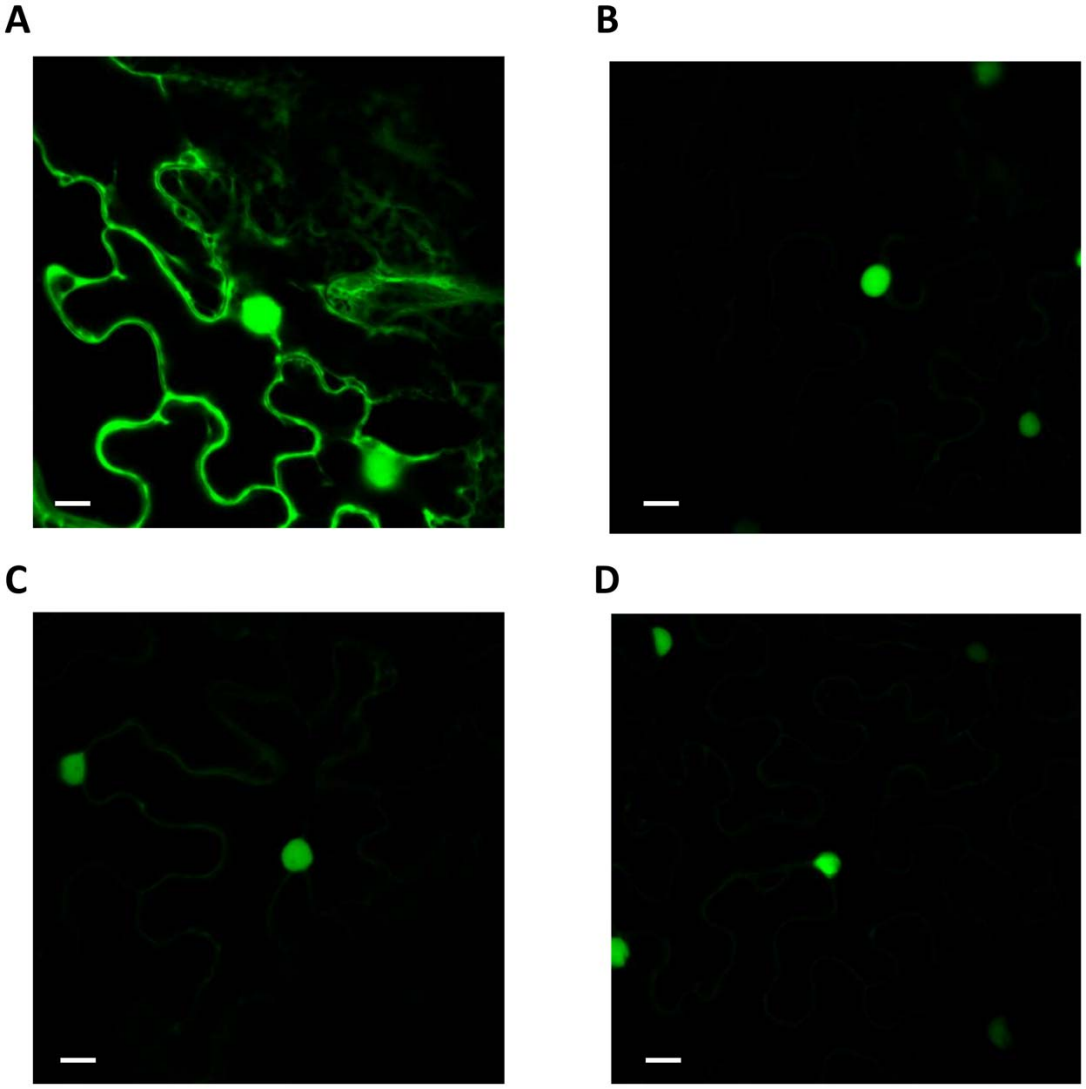
Cellular localisation of *Vaccinium* GFP-CBF protein fusions. Confocal microscopy images of *Vaccinium* GFP-CBF proteins (B–D) compared to a free GFP control (A). GFP fusions to CBFs from *V. myrtillus* (B), *V. uliginosum* (C) and *V. vitis-idaea* (D). White scale bars represent 22 µm.

**Figure 8:**
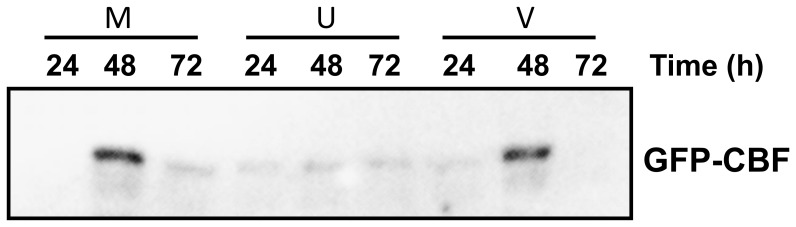
Protein expression of Vaccinium GFP-CBF fusions. Western blot analysis of proteins extracted from *Vaccinium* GFP-CBF expressed in *Nicotiana benthamiana* 24, 48 and 72 hours after infiltration. “M”, “U” and “I” refer to *V. myrtillus*, *V. uliginosum* or *V. vitis-idaea* CBF, respectively.

In order to test if there were differences in levels of proteins or targeting to the nucleus when expressing the three *Vaccinium* CBFs we produced GFP tagged versions that could be visualised in cells using fluorescence microscopy, and levels of protein determined by western blot analysis using an antibody to GFP. We tested these parameters by transiently expressing these constructs in *Nicotiana benthamiana*. Firstly, however, confirmation that the GFP tag did not affect the differential activation of target gene expression by the *Vaccinium* CBFs was required. We therefore co-expressed each of the *Vaccinium* CBF-GFP fusions together with a construct which consisted of 4 copies of the CRT/DRE (CBF-binding motif) fused to luciferase [26]. The effects of expression of *Vaccinium* CBF upon CRT/DRE-controlled LUC expression was determined by comparing LUC expression obtained by co-expressing a free-GFP control. As shown if Figure 6, the *V. myrtillus* CBF-GFP construct was able to induce LUC expression to a higher relative level than *V. myrtillus*/*V. vitis-idaea* CBFs, mimicking the effect of non-tagged CBFs in transgenic *Arabidopsis* (Figures 2 and 3). Interestingly, in transient expression in *Nicotiana benthamiana*, both the *V. vitis-idaea* and *V. uliginosum* CBFs were able to transactivate the DRE/CRT, unlike in stable transgenic *Arabidopsis*. The levels of transactivation in *Nicotiana benthamiana*, however, were not as high as for *V. myrtillus*, with *V. vitis-idaea* and *V. uliginosum* CBFs inducing to only roughly 30 and 50% of the levels seen with *V. myrtillus* CBF, respectively. To test for localisation of the protein fusions in the cell we imaged the GFP fusions using confocal microscopy. As shown in Figure 7, the localisation of all three CBF-GFP fusions was exclusively nuclear. As expected the free GFP control was localised in both the cytosol and the nucleus. We tested protein expression of all 3 CBFs 24, 48 and 72 h after infiltration, by extracting proteins and performing western blot analysis using a GFP polyclonal antibody. These data showed that there was a peak of CBF-GFP levels after 48 h for both *V. myrtillus* and *V. vitis-idaea*, and levels were very similar between the two (Figure 8). However for *V. uliginosum* the levels of protein were much lower, and did not show any clear peak of expression.

## Discussion

Our main aim was to assess the potency of CBF/DREB1 transcription factors from three Arctic plant species from the genus *Vaccinium*, namely *V. myrtillus*, *V. uliginosum* and *V. vitis-idaea*, as these species have the ability to tolerate significantly low temperatures [16], [17], [18]. To determine whether the predicted CBF protein sequences from *V*. *myrtillus*, *V. uliginosum* and *V. vitis-idaea* might reveal differences that could be correlated to altered transcription factor activity, we cloned a single CBF coding from each species. It is important to bear in mind that the three *Vaccinium* species are likely to have multiple *CBF* genes as do other plant species. None of the three *Vaccinium* species has had their genomes sequenced yet, so we cannot yet gauge the complexity of the *CBF* gene families in these three species. It is important, therefore, to bear in mind that conclusions regarding differences in CBF activity in the three species in a environmental context must be circumspect, as other (not revealed) *Vaccinium* CBF proteins may have totally different activity. Lining up the three *Vaccinium* CBF protein sequences with existing CBF/DREB1 sequences from *Arabidopsis* [9], another accession of *V. vitis-idaea* from China [38] and *V. corymbosum* [13] allowed us to compare three important protein domains for these proteins, namely the AP2/EREBP signature domain [40], the DSWAR region [40] and the COOH region [42]. In *Arabidopsis* the AP2/EREBP signature domain has been shown to be important to binding of CBF to its cognate DNA element, the DRE/CRT [40]. As can be seen in figure 1 the AP2/EREBP, which in all three *Arabidopsis* CBF genes is “PKKPAGRKKFRETRHP”, shows two changes in the *Vaccinium* genus namely “PKKRAGRKKFKETRHP”. These proline to arginine and arginine to lysine substitutions were found in all five *Vaccinium* sequences. As it had been previously demonstrated that *V. corymbosum* CBF could activate *COR* gene expression in transgenic *Arabidopsis* [13] and *Vaccinium* [14], it is clear that these changes do not inhibit binding of CBF to the DRE/CRT DNA motif. Similarly for the DSAWR sequence, there was one alanine to valine substitution that was common in all five *Vaccinium* sequences in comparison to *Arabidopsis*. By the same logic, this substitution cannot be responsible for inhibiting binding to the DRE/CRT element. However, strikingly, the sequence for *V. vitis-idaea* from the Swedish accession we collected, had one substitution not present in any of the other sequences, be they *Arabidopsis* or *Vaccinium*. This was an arginine to glutamine. This “DSVWR” to “DSVWQ” change was not found in the *V. vitis-idaea* accession from China [38]: clearly resulting from a polymorphism in the Northern Swedish population. Whilst the DSVWR motif is highly conserved in plant CBF/DREB1s [40] it has not, to our knowledge, been demonstrated to be essential for binding to the DRE/CRT motif. However, the amino acid substitution in our Northern Sweden accession of *V. vitis-idaea* might be responsible for reduced binding activity. Comparing the COOH regions, which are responsible for transcriptional activity [42], whilst there were numerous differences in sequences between *Arabidopsis* and *Vaccinium* CBF sequences, there was only a single difference between the five *Vaccinium* sequences. This difference was in a single species, *V. corymbosum*, where a leucine was present where in all four other *Vaccinium* species an isoleucine was present. Interestingly, the *V. corymbosum* sequence was identical to the *Arabidopsis* sequence at this point. Given that it has been demonstrated that all three *Arabidopsis* CBF sequences, and *V. corymbosum* CBF can activate *COR* genes in transgenic *Arabidopsis* [9], [13] it was a formal possibility that in the other three *Vaccinium* species the leucine to isoleucine substitution was sufficient to render them incapable of transactivation. However, the similarity in properties between leucine and isoleucine rendered this possibility relatively unlikely.

To empirically test these sequence-based predictions, we overexpressed CBF cloned from *V*. *myrtillus*, *V. uliginosum* and *V. vitis-idaea* all collected in Northern Sweden. As can be seen in Figure 2A transgenic lines were obtained for the three different *Vaccinium* CBF sequences. Interestingly, comparison of the levels of *CBF* overexpression showed than on average, the levels of expression were lower for *V*. *myrtillus* than the other two species. This implied some sort of fitness penalty for *CBF* overexpression for this species, selecting against highest expressers. When the expression of CBF target *COR* genes *KIN2*, *GOLS3* and *LTI78* (Figure 2B, 2C and 2D, respectively) was measured, it could be clearly seen that on average, only *V*. *myrtillus* CBF expression resulted in greatly elevated *COR* gene expression compared to wild type. For *V. corymbosum* CBF it had been reported that it was incapable of inducing *KIN2*, *GOLS3* and *LTI78* when overexpressed in *Arabidopsis*, but was able to induce *COR15A* and *COR414* [13]. As seen in Figure 3, *V*. *myrtillus* was also able to induce both of these *COR* genes. This implies that the selectivity shown by *V. corymbosum* CBF [13] is not a feature of *V*. *myrtillus* CBF. How such selectivity could occur in *V. corymbosum* is not clear, but this property must reside in the differences between the CBF protein sequences in the two species. Comparison between Figure 2A and Figures 2B, 2C and 2D and Figure 3 show that the level of *V*. *myrtillus* CBF overexpression did not correlate with levels of *COR* gene expression. This implies that the levels of *CBF* transcripts do not correlate with CBF protein level/activity directly, and implies posttranslational control. It is very clear that the CBF sequences from *V. uliginosum* and *V. vitis-idaea* were very ineffective at inducing the expression of *COR* genes, despite very high levels of *CBF* transcripts (Figures 2 and 3). In the case of *V. vitis-idaea* this could be due to poor binding to the CRT/DRE element if the amino acid substitution in the DSAWR domain described above was significant. However, in the case of *V. uliginosum* the prediction would be that it would bind CRT/DRE as well as CBF from *V*. *myrtillus* and *V. corymbosum*. Similarly, the poor induction of *COR* genes by *V. uliginosum* and *V. vitis-idaea* CBF could not be due to poor transactivation as the COOH domains were identical to *V*. *myrtillus* CBF (Figure 1). It was therefore possible that the differences in potency were as a result of differences in posttranscriptional regulation of *V. uliginosum* and *V. vitis-idaea* CBF. Posttranscriptional regulation could include differences in efficiency of targeting to the nucleus or levels of protein.

To test these possibilities directly, we produced GFP-tagged versions of CBF from the three *Vaccinium* species, and transiently expressed them in *Nicotiana benthamiana*. As the GFP tag might affect the function of the CBF we firstly tested the ability of all three GFP fusions to induce a *DRE/CRT::LUC* construct by co-expressing them. As can be seen in Figure 6, the pattern of activation seen in stable *Arabidopsis* expressing non-tagged CBF was replicated: the *V*. *myrtillus* CBF was far more effective at activating expression *via* DRE/CRT promoter motif than the CBFs from the other two species. Interestingly, in the transient expression system *V. vitis-idaea* CBF was capable of transactivating *CRT/DRE::LUC* (Figure 6), even though it had been unable to induce *COR* genes in transgenic *Arabidopsis* (Figures 2 and 3). There are several possibilities for this difference, for example the levels of overexpression in *Nicotiana benthamiana* might be sufficiently greater to overcome any potential reduced binding to CRT/DRE binding-efficiency as a result of the “DSVWR” to “DSVWQ” change (see above). We examined the cellular localisation of the GFP-CBF fusions using confocal microscopy (Figure 7). Our results showed that there was no difference in the localisation of all three CBF GFP fusions: all were discreetly localised to the nucleus. To determine the relative levels of the three proteins, we performed western blot analyses on proteins extracted from *Nicotiana benthamiana* tissue in which the GFP fusions were expressed. We analysed the levels of protein expression 24, 48 and 72 h after infiltration. Both *V. vitis-idaea* and *V*. *myrtillus* CBF proteins showed the same behaviour: peaking in expression at 48 h, with relatively high levels of expression (compared to 24 and 72 h timepoints, and compared to *V. uliginosum*). The situation with *V. uliginosum* was considerably different, with relatively low levels of protein, and no appreciable peak. Thus, the lack of potency for *V. uliginosum* at inducing *COR* gene expression (Figures 2 and 3) or transactivating the DRE/COR element (Figure 6) could be due to low protein levels. Given that the constructs we used were identical apart from the three coding regions, it is highly unlikely that this low level of protein is due to lower levels of translation, thus we conclude that the *V. uliginosum* CBF is relatively unstable compared to CBF from the other two *Vaccinium* species. This difference is most likely due to differences in protein sequence *e.g.* presence of sites for ubiquitination in the *V. uliginosum* sequence. For instance, lysine78 in the *V. uliginosum* CBF sequence is not present in the other two *Vaccinium* species. Whether this is a site for translational modification *e.g.* polyubiquitination leading to proteosomal degradation would be an interesting topic for future study. On the other hand, the lack of a difference in protein expression (Figure 8) and targeting (Figure 7) for *V. vitis-idaea* compared to *V*. *myrtillus* does not explain the inability of the former to induce *COR* genes (Figures 2 and 3) or fully transactivate DRE/CRT (Figure 6). Along with the fact that the sequence data render the likelihood of poor binding to DRE/CRT or ineffective transactivation domain low, one can only surmise that the difference is due to reduced ability to interact with other components of the transcriptional machinery *e.g.* Mediator. In such a scenario, the amino acid differences between *V. vitis-idaea* and *V*. *myrtillus* CBF might be responsible, and it would be interesting to test mutations of *V. vitis-idaea* CBF towards the *V*. *myrtillus* sequence to see if activity can be rescued.

The ability of *V*. *myrtillus* CBF to activate *COR* gene expression in transgenic *Arabidopsis* indicated the possibility that there would be an increase in the freezing tolerance of these lines. This was tested and it was confirmed that transgenic *Arabidopsis* overexpressing *V*. *myrtillus* CBF was more freezing tolerant than either wild type or transgenic *Arabidopsis* expressing CBF from the other two *Vaccinium* species. Also importantly, the growth phenotype associated with CBF overexpression, dwarfism, was also only manifest when *V*. *myrtillus* was overexpressed.

*V. myrtillus* in the Arctic can only survive the winter under a deep insulating snow cover: loss of this cover results in extreme frost damage [43]. *V. vitis-idaea* and *V. uliginosum* can both survive under much shallower, and thus colder, snowpacks in more exposed parts of the natural landscape. It is thus clear that *V. vitis-idaea* and *V. uliginosum* have evolved relatively greater freezing tolerance with respects to *V. myrtillus*. If the behaviour of the *Vaccinium* CBFs observed in *Arabidopsis* and *Nicotiana* represent the behaviour in the host genetic background, this would suggest the higher level of freezing tolerance found in *V. vitis-idaea* and *V. uliginosum* is not CBF-based, but resulting from another pathway/trait. One has to apply caution, however, when trying to extrapolate CBF function in the native species using data based upon prediction of CBF function by expressing in a heterologous system (*Arabidopsis* and *Nicotiana*) and behaviour of the CBF proteins could be different in their natural genetic backgrounds. Also, as in *Arabidopsis*, the CBF protein of the three *Vaccinium* species may be encoded by a multigene family, and more potent versions of CBF found in the *V. uliginosum* and *V. vitis-idaea* genomes. In the future, taking a whole genomic approach in these *Vaccinium* species *e.g.* full genome sequencing and assembly, will answer these questions. It will be important along with this to develop proteomic and transcriptomic platforms for these species to fully elucidate the molecular basis of freezing tolerance in these remarkable species.

## Supporting Information

Figure S1 **Expression of CBF in V. myrtillus, V. uliginosum and V. vitis-idaea.** Relative CBF transcript abundance was measured in either *V. myrtillus*, *V. uliginosum* or *V. vitis-idaea* (“M”, “U” and “I”, respectively). ”A” corresponds to ambient and “C” is a 2 h treatment at 5°C. Data was normalised using beta-tubulin expression. Error bars represent RQ_MIN_ and RQ_MAX_ and constitute the acceptable error level for a 95% confidence level according to Student’s t-test [31].
(TIF)
