# Supplementary figures and images for: A C-Repeat Binding Factor Transcriptional Activator (CBF/DREB1) from European Bilberry (Vaccinium myrtillus) Induces Freezing Tolerance When Expressed in Arabidopsis thaliana
Source: PLoS One. 2013 Jan 17;8(1):e54119. doi: 10.1371/journal.pone.0054119 (PMC3547970; doi:10.1371/journal.pone.0054119)

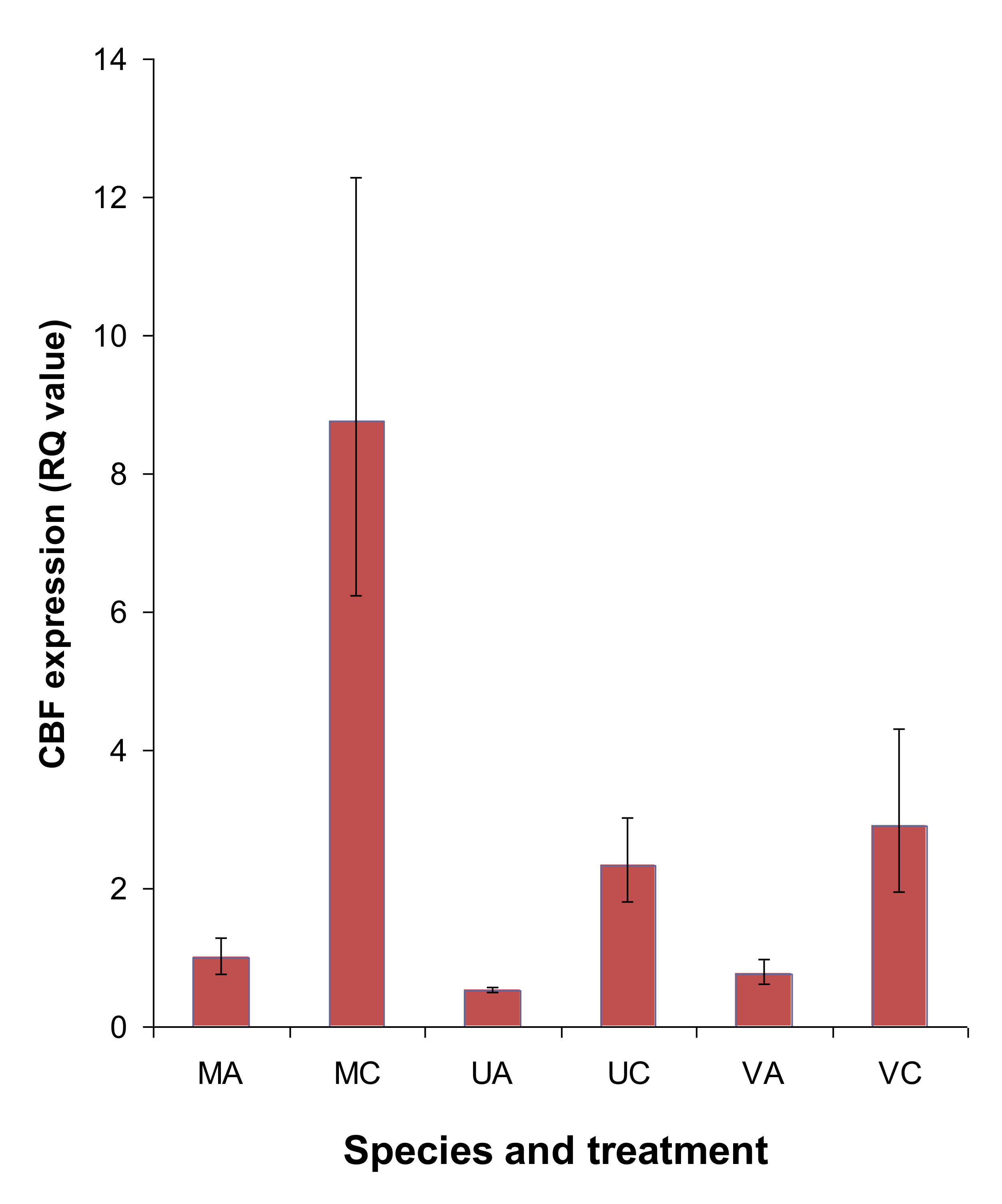

Supplement: Figure S1 — Expression of CBF in V. myrtillus, V. uliginosum and V. vitis-idaea. Relative CBF transcript abundance was measured in either V. myrtillus, V. uliginosum or V. vitis-idaea (“M”, “U” and “I”, respectively). ”A” corresponds to ambient and “C” is a 2 h treatment at 5°C. Data was normalised using beta-tubulin expression. Error bars represent RQMIN and RQMAX and constitute the acceptable error level for a 95% confidence level according to Student’s t-test [31]. (TIF) [file pone.0054119.s001.tif]
